# Genetic diversity study and molecular marker development for economic traits in Korean pine based on RAD-Seq

**DOI:** 10.3389/fpls.2026.1887054

**Published:** 2026-07-21

**Authors:** Pingyu Yan, Guang Li, Lei Zhang, Zixiong Xie, Zhen Miao, Chao Wang, Mengyuan Liu, Ruike Song, Libin Wang, Jiaxing Wang, Xiaotian Miao, Xiaoyu Tian, Junfei Hao, Zhixin Li, Hanguo Zhang

**Affiliations:** 1 State Key Laboratory of Tree Genetic and Breeding, Northeast Forestry University, Harbin, China; 2 School of Forestry, Xinyang Agriculture and Forestry University, Xinyang, China

**Keywords:** genetic diversity, GWAS, KASP, *Pinus koraiensis*, RAD-seq, SNP

## Abstract

To reveal the genetic status of Korean pine germplasm resources, explore SNP loci associated with important economic traits of Korean pine, and develop breeding markers, the economic traits of 100 clones from 7 populations (planted in 6 seed orchards) were observed and reduced genome sequencing on the basis of Restriction-site associated DNA sequencing (RAD-Seq) was performed. Three models from the R software package “rMVP” were used for genome-wide association studies (GWAS). A total of 14,463,334 Single nucleotide polymorphisms (SNP) markers were obtained by variant detection and used for subsequent genetic analysis and GWAS. The average linkage disequilibrium- decay (LD- decay) of the SNP pairs varied from 3.383 (Weihe) to 96.245 (Lushuihe). The population with the highest Shannon index was Weihe (0.377) and the lowest was Lushuihe (0.331). The Fst between populations ranged from 0.0183 to 0.0516. A total of 16 traits were associated with 1692 SNP loci. On the basis of the GWAS results, Kompetitive allele-specific PCR (KASP) markers were developed and validated using 119 natural Korean pine plus trees. A total of 4 KASP markers were developed for cone number and cone weight. The genetic diversity of Korean pine populations is similar, and there is little differentiation between populations. Several SNPs related to economic traits, such as CN, have been obtained through GWAS. The developed KASP markers promote molecular marker-assisted selection (MAS) in breeding programs, improving the efficiency and accuracy of genetic improvement in Korean pine.

## 1 Introduction

Korean pine (*Pinus koraiensis*) is a valuable edible nut and oilseed tree species in northeastern China (Du et al., 2017), and it is also an important source of pine nut products worldwide, with significant economic value (Yang et al., 2009). However, Korean pine grows slowly and has a long juvenile period (Feng et al., 2006), which poses significant challenges for its breeding. The main reason is that its fruit-bearing capacity manifests itself only after a long juvenile period, resulting in the current breeding progress of Korean pine remaining at the 1st generation and the reconstructed 1.5th generation seed orchard (Kele et al., 2023). The level of fruit-bearing capacity not only affects the economic benefits of nut orchards for pine nut production, but also serves as an essential factor limiting the production efficiency of seed orchards, which are critical sites for seed propagation. Therefore, molecular breeding techniques are highly important for accelerating the breeding process of Korean pine.

Conifers are known for their large genomes, especially pines, whose genome sizes are even larger than those of other gymnosperms (17–35 Gb). For example, the genome size of *Pinus tabulaeformis* is as high as 25.4 Gb (Murray, 1998; Niu et al., 2022). Genotyping of conifers is limited by their large and highly repetitive genomes, whereas phenotyping is hampered by their long-life-cycle and heterogeneous living environments (De La Torre et al., 2019; Chen et al., 2021b). Thus, the genetic code of Korean pine remains unknown. For tree species lacking a reference genome, such as Korean pine, genotyping-by-sequencing (GBS) is considered the most promising technological choice (Altshuler et al., 2000; Elshire et al., 2011). In recent years, with the rapid development of high-throughput sequencing technology, the specific-locus amplified fragment sequencing (SLAF-seq) method has emerged. This technique allows the sequencing of specific genomic regions, resulting in many polymorphic genetic markers that can adequately represent the whole genome information of the target species (Miller et al., 2007). Restriction-site associated DNA sequencing (RAD-Seq) is a strategy known as the “genome complexity reduction” approach (a form of GBS), which has proven particularly useful in nonmodel species because of its combination of low cost and high throughput (Baird et al., 2008; Rowe et al., 2011). RAD-Seq has been applied to various aspects of genetic/genomic research in more than 20 species without reference genomes, including efficient marker development (Scaglione et al., 2012; Pegadaraju et al., 2013; Pujolar et al., 2013), genetic and comparative mapping (Baxter et al., 2011; Kakioka et al., 2013; Yang et al., 2013), high-resolution gene/QTL mapping (Chutimanitsakun et al., 2011; Pfender et al., 2011; Hegarty et al., 2013), phylogenetic/phylogeographic analysis (Emerson et al., 2010; Nadeau et al., 2013), and genome-wide association studies (GWASs) (Hecht et al., 2013).

The application of GWAS in plant science has constituted a significant advancement in the field of genetics and genomics research in recent years. There have been notable advances in the field of plant genome sequencing (Werner, 2010; Clarke et al., 2016; Li et al., 2018). This has led to the development of techniques that can rapidly and cost-effectively detect genotype data from a large number of individuals. This provides the necessary technical support for the implementation of GWAS in plants. GWAS employs statistical methods to identify associations between genomic polymorphisms (e.g., SNPs, insertions and deletions, and structural variations) and phenotypic variations (Xiao et al., 2022), thereby greatly facilitating the analysis of genetic structures related to complex traits (Rebolledo et al., 2016). This includes crops such as *Zea mays* L., *Oryza sativa indica*, *Gossypium hirsutum*, and *Glycine soja* (Buckler et al., 2009; Huang et al., 2010; Fang et al., 2017; Leamy et al., 2017; Liu and Yan, 2019), thereby increasing the efficiency of genomic selection. In the absence of a complete reference genome for Korean pine, the identification of single nucleotide polymorphisms (SNPs) or other genetic markers associated with target traits through GWASs based on techniques such as RAD-Seq or GBS can still facilitate the development of trait-related molecular markers. These markers can be directly applied to molecular marker-assisted selection (MAS), accelerating the genetic improvement process of crops and trees and thereby increasing breeding efficiency. KASP (Kompetitive Allele-Specific PCR), a proprietary genotyping technology developed by LGC Biosearch Technologies, employs allele-specific oligonucleotide extension and fluorescence resonance energy transfer (FRET) for signal generation (Kumpatla et al., 2012). Over the past decade, this technology has been extensively employed by plant breeders for MAS, supplanting gel-based molecular marker analyses such as simple sequence repeat (SSR) markers (Makhoul et al., 2020).

This study for the first time employed RAD-Seq to sequence 100 breeding parents from six Korean pine seed orchards (Bohai, Hegang, Lushuihe, Tieli, Weihe, Sanchazi) in Northeast China (Heilongjiang and Jilin Provinces). We aimed to conduct a genome-wide association study (GWAS) on yield-related economic traits and screen yield-associated SNPs. The identified SNPs were further converted into KASP markers and validated in natural populations. The obtained SNPs not only elucidate the genetic structure of Korean pine germplasm resources but also provide new insights into the genetic basis of complex quantitative traits in this species. Fruit-related KASP markers have the potential to shorten the breeding cycle of Korean pine and facilitate the selection of high-yield germplasm resources.

## 2 Materials and methods

### 2.1 Plant materials

**Figure 1:**
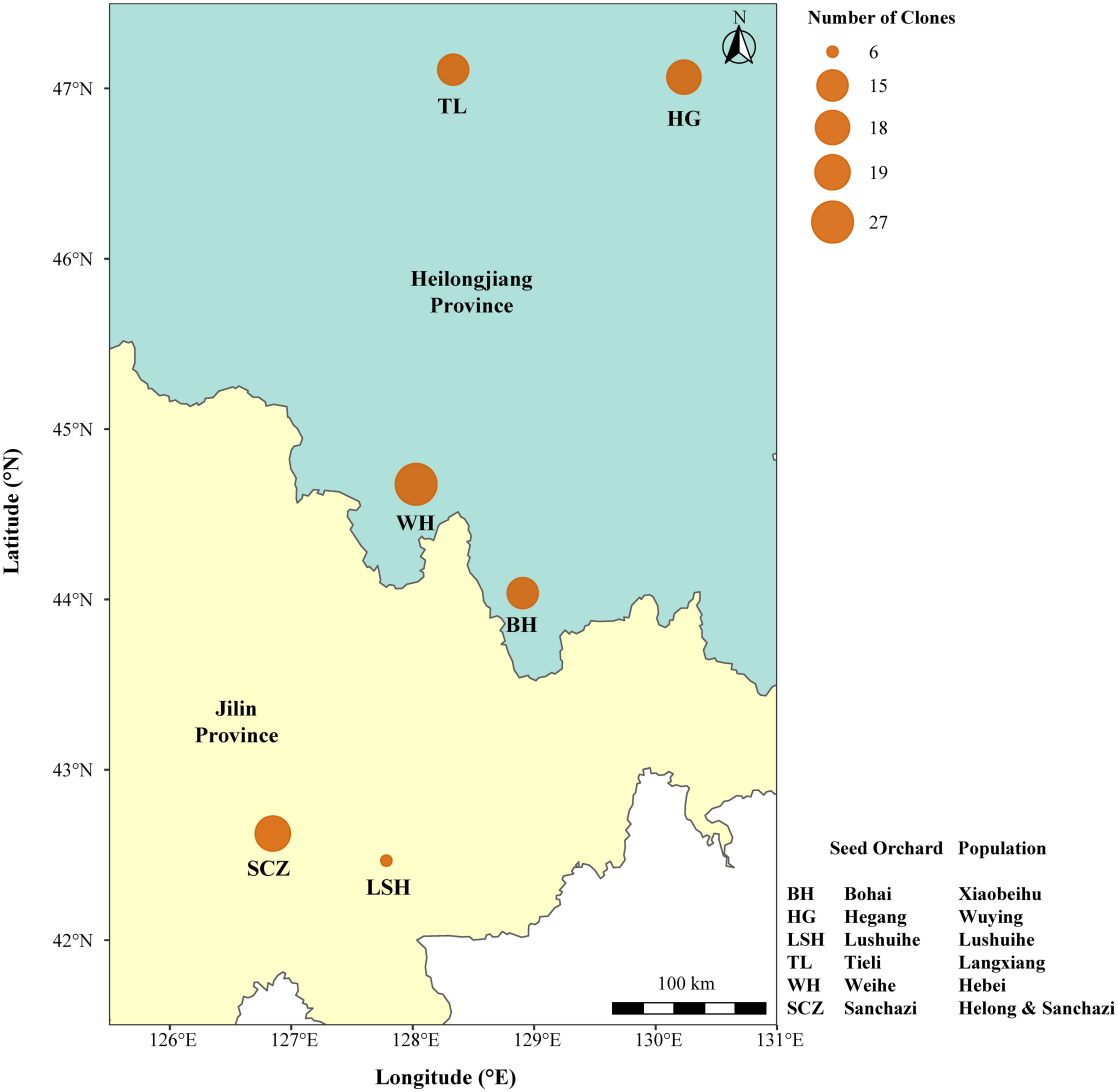
Geographic Distribution of Korean Pine Seed Orchards in Northeast China.

A total of 100 clones, sourced from seven natural populations of Korean pine, were planted in 6 clone seed orchards of Korean pine in Northeast China. Each seed orchard corresponds to one native source population, except the Sanchazi seed orchard, which contains two populations (Helong and Sanchazi) (**Figure 1**). All clones were grafted onto 4-year-old rootstocks, and consistent cultivation and field management regimes were implemented within each seed orchard. At the time of leaf tissue sampling in 2023, the grafted clones were 40–50 years old, with stable fruit-bearing performance and fully developed reproductive traits suitable for yield-related phenotypic investigation.

### 2.2 Methods

#### 2.2.1 Phenotypic data

A study was conducted on 100 clones of Korean pine, with an examination of various phenotypic traits, including cone number (CN), cone weight (CWE), cone length (CL), cone width (CW), the cone length-to-width ratio (CL/CW), seed length (SL), seed width (SW), the seed length-to-width ratio (SL/SW), the seed kernel weight (SKWE), the seed coat weight (SCWE), the seed weight per cone (SWE), the seed weight per thousand (SWET), the seed yield rate (SWE/CWE), the kernel yield rate (SKWE/SWE), the empty shell rate (ER), and the seed number per cone (SN). A completely randomized design (CRD) was implemented for clone layout across all six seed orchards. To minimize sampling bias and guarantee phenotypic accuracy, five healthy, disease-free and normally fruiting ramets per clone were randomly chosen as biological replicates. All cone and seed yield-related phenotypic traits were quantified systematically during three consecutive growing seasons from 2020 to 2022.

Furthermore, an investigation was conducted on the CN and CWE of 119 natural Korean pine plus trees from Xiaobeihu Forest Farm over the period from 2021–2023. For phenotypic comparison among different KASP genotypes, linear mixed model (LMM) was applied to eliminate environmental noise before statistical analysis. Forest age, site condition and stand management level were set as random effects, and genotype was defined as fixed effect. The adjusted BLUP values of CN and CWE after environmental correction were used for multiple comparisons instead of raw phenotypic data.

The following definitions are provided for the terms used in this study:

CN: The mean number of cones produced by each ramet.
CWE (g): The weight of a single cone.
CL (mm): The distance from the tip to the base of a cone.
CW (mm): The widest distance across a cone.
CL/CW: The ratio of cone length to cone width.
SL (mm): The distance from the tip to the base of a seed.
SW (mm): The widest distance across a seed.
SL/SW: The ratio of seed length to seed width.
SKWE (g): The total weight of all seed kernels within a single cone.
SCWE (g): The total weight of all seed coats within a single cone.
SWE (g): The total weight of all seeds within a single cone.
SWET (g): The total weight of 1000 seeds.
SWE/CWE: The percentage of the total seed weight within a single cone relative to the weight of the cone itself.
SKWE/SWE: The percentage of the total seed kernel weight within a single cone relative to the total seed weight.
ER (%): The percentage of empty shells among all seeds.
SN: The number of seeds within a single cone.

To eliminate inter-annual environmental variation of phenotypic traits across 2020–2022, best linear unbiased prediction (BLUP) values of each clone were calculated using lme4 package in R software, with clone as fixed factor and year as random factor. BLUP-adjusted phenotypic values rather than raw trait data were used for subsequent GWAS correlation and association analysis. For 119 natural plus trees with phenotypic data collected from 2021–2023, mixed model BLUP correction was also conducted to remove age and site noise before genotype comparison.

#### 2.2.2 RAD-Seq

One-year-old needles from 100 clones of Korean pine were collected, and DNA was extracted via the CTAB method (Porebski et al., 1997). The quality of the extracted genomic DNA was verified via two methods: (1) assessment of DNA degradation and contamination on a 1% agarose gel and (2) measurement of the DNA concentration via an ND-2000 spectrophotometer (NanoDrop Technologies). Only high-quality DNA samples, with an OD260/280 ratio between 1.8 and 2.0 and an OD260/230 ratio of at least 2.0, were used for the construction of sequencing libraries. A total of 0.5 μg of DNA from each sample was used as the material for the DNA library preparation. The sequencing libraries were generated via the TruSeq Nano DNA HT Sample Prep Kit (Illumina, USA) in accordance with the manufacturer’s instructions, with index codes appended to each sample. In summary, the genomic DNA samples (0.1-1 μg) was digested with the restriction enzyme NlaIII, resulting in fragments of approximately 350 base pairs. The DNA fragments subsequently underwent end-repair, A-tailing, and ligation with full-length adapters for Illumina sequencing, followed by polymerase chain reaction (PCR) amplification. After purification of the PCR products via the AMPure XP system, the size distribution of the libraries was analysed via the Agilent 2100 Bioanalyzer, and quantitation was performed via RT–PCR (3 nM). The paired-end DNA-seq libraries were sequenced via the Illumina NovaSeq system at Shanghai Majorbio Biopharm Technology Co., Ltd.

Raw RAD reads were filtered with sequencing depth threshold ≥5× for each locus; predicted NlaIII enzyme-cut fragments were restricted to 300–400 bp to standardize library construction. After variant calling, preliminary SNP quality control was performed before downstream analysis: markers with missing rate >10% or minor allele frequency (MAF) <0.05 were removed to avoid false variation signals, consistent with the filtering criteria applied in GWAS analysis.

#### 2.2.3 Development of KASP

One-year-old needles were collected from 119 natural Korean pine plus trees from Xiaobeihu Forest Farm. Genomic DNA was extracted, and its quality was tested in accordance with the methodology outlined in section 1.2.2. The extracted DNA was diluted to 50 ng/μl to be suitable for subsequent use.

Primers were designed and synthesized based on the basis of the read sequence containing the associated SNP, comprising two allele-specific forward primers and one common reverse primer. The 3’ terminal bases of the two specific forward primers were the variant bases at the SNP site, and universal fluorescent labels were added to the 5’ ends:

F1 (FAM): GAAGGTGACCAAGTTCATGCT
F2 (VIC): GAAGGTCGGAGTCAACGGAT

All the abovementioned primers were synthesized by Sangon Biotech (Shanghai) Co., Ltd.

The synthesized primers were diluted to 10 μM by TE (pH 8.0) and then mixed into a primer mixture at a ratio of 1:1:3 for forward primer 1, forward primer 2, and reverse primer.

The PCR system employed a 5 μl reaction volume, which comprised the following components: 2.5 μl of HiGeno2×ProbeMixA (JasonGen Biological Technology Co., Ltd., Beijing), 1.25 μl of primer mixture, and 1.25 μl of DNA sample. The touchdown PCR was conducted on a CFX Connect™ Real-Time System (Bio-Rad, USA), and the reaction protocol is presented in **Table 1**.

**Table 1:** The reaction protocol used for touchdown PCR.

Step	Description	Temp	Time	NO. of cycle
1	Activation	95 °C	10 min	1
2	Denaturation	95 °C	20 sec	10
	Annealing/Elongation	61 °C, decreasing by 0.6 °C per cycle to a final temperature of 55 °C	60 sec	
3	Denaturation	95 °C	20 sec	27
	Annealing/Elongation	55 °C	60 sec	
4	Read	25 °C	30 sec	1

#### 2.2.4 Statistical analysis

Low-quality raw reads (with an average Phred score<20), including fragments contaminated with adapters and unrecognizable nucleotides (N bases>10), were subjected to trimming or discarding via Fastp v0.19.6 (Chen et al., 2018). The identification of variants was conducted via Stacks v2.3 (Catchen et al., 2013). The population linkage disequilibrium (LD) decay distance (*r*²) was calculated via PopLDdecay (Zhang et al., 2019). A neighbor-joining (NJ) evolutionary tree was constructed through 1,000 iterations via FastTree v2.1.11 (Price et al., 2010). The population structure was analysed on the basis of single-nucleotide polymorphisms (SNPs) via ADMIXTURE v1.3.0 (Alexander et al., 2009). Clustering was performed on the assumption that the number of subpopulations (K value) ranged from 1–20. The optimal number of subpopulations was determined by identifying the K value corresponding to the lowest cross-validation error (CV error). Genetic parameters, principal component analysis (PCA), and an evaluation of kinship among individuals (Ani et al., 2010) were performed via Plink (Purcell et al., 2007).

GWASs for the target traits were conducted via the general linear model (GLM), the mixed linear model (MLM), and the fixed and random model Circulating Probability Unification (FarmCPU) from the R package “rMVP” (Yin et al., 2021). GLM only incorporates population structure (Q matrix) as fixed effects, ignores polygenic relatedness. Prone to abundant false positives in populations with strong genetic relatedness. MLM combines Q matrix (fixed population structure) and kinship matrix K (random polygenic effect). Effectively eliminates false positives caused by both population stratification and clonal relatedness, selected as the core model for this study. FarmCPU adopts an iterative strategy of fixed and random models for genome-wide association analysis. The standard model formula of MLM is:


y=Xβ+Zu+e

Where:

*y*: Vector of adjusted phenotypic BLUP values for each clone;
*X*: Fixed effect matrix (including population structure Q matrix and SNP marker genotype);
*β*: Vector of fixed effect coefficients to be estimated;
*Z*: Random effect incidence matrix for individual genotypes;
*u*: Vector of polygenic random effects, following *u*∼*N* (0, *Kσ*2 *u*);
*K*: Kinship matrix among all clones.
*e*: Residual error vector, following *e*∼*N* (0, *Iσ*2 *e*).

Population structure was pre-estimated via ADMIXTURE v1.3.0 with K ranging from 1 to 20, and cross-validation (CV) error for each K was calculated to determine the optimal subpopulation number. The minimum CV error appeared at K = 1, indicating no distinct genetic subgroups among the 7 Korean pine populations. To fully correct weak population stratification signals, the top three PCs derived from PCA were still incorporated as fixed covariate matrix Q in GLM, MLM and FarmCPU models, together with IBS kinship matrix K as random covariate to eliminate false positive associations induced by population genetic differentiation. The CV error curve of K = 1–20 supported the rationality of covariate selection in subsequent GWAS. The identity-by-state (IBS) kinship matrix *K* was constructed using filtered high-quality SNP markers in TASSEL v5.0. The IBS algorithm calculates pairwise genetic similarity between every pair of clones by counting shared identical alleles across all valid markers, generating a symmetric n×n kinship matrix (n = 100 clones) to quantify pairwise genetic relatedness for random effect correction in MLM. Two independent significance thresholds were adopted to screen reliable trait-associated SNPs to avoid false positives: (1) Bonferroni correction threshold: P < 1/N_SNP = 2.4×10^-^^6^, where N_SNP represented the total filtered high-quality SNPs; (2) empirical threshold from 10,000 genome-wide permutation tests per trait, defined as the 95th quantile of permutation-derived minimal P-values. Only SNPs simultaneously satisfying both thresholds were regarded as stable high-confidence association loci. For ER and CL/CW with obvious λ deviation, conditional GWAS was further implemented by fixing significant lead SNPs as covariates to exclude redundant linkage loci; loci failing conditional GWAS validation were discarded from final association sets to reduce false positive noise. To verify whether this threshold was excessively stringent, we conducted 10,000 genome-wide permutation tests for each phenotypic trait: clone genotypes were randomly shuffled against BLUP phenotypic values, and the minimum genome-wide P-value from each permutation was recorded to generate an empirical null distribution. The 95th quantile of permutation-derived P-values was defined as the empirical significance cutoff. Loci detected by both Bonferroni and permutation thresholds were regarded as stable, high-confidence seed-yield associated markers.

The statistical description, analysis of variance (ANOVA), and correlation analysis of the phenotypic data were performed via SPSS v27 (Salcedo and McCormick, 2015).

## 3 Results

### 3.1 Sequencing quality and variant detection

The RAD-Seq results of 100 clones of Korean pine revealed the following (**Table 2**): an average of 26,106,340 high-quality reads per sample, with a total base count of 3,654,887,572 for high-quality sequencing data. These findings were obtained following quality trimming. The mean Q30 value was 90.08%, while the mean GC content was 37.06%. A total of 5,368,630 enzyme-cut fragments were identified, with an average of 4,392,602 enzyme-cut sites per sample and an average sequencing depth of 6.275. A total of 14,463,334 SNPs were identified through variant detection, with an average of 4,392,602 SNPs detected per sample. The SNPs were subsequently employed in genetic diversity and GWAS.

**Table 2:** Sequencing quality and SNP statistics.

Metrics	Mean	SD	Max	Min	Med
Clean reads	26,106,340	4,109,334	40,001,776	16,891,452	26,061,744
Clean base	3,654,887,572	575,306,821	5,600,248,640	2,364,803,280	3,648,644,160
Q30 (%)	90.08	2.08	93.57	80.83	90.4
GC (%)	37.06	8.26	52.96	7.01	37.87
Present tags	4,392,602	6,310,658	483,933	4,520,624	1,019,544
Present SNPs	716,935	1,127,165	59,949	724,461	192,199
Mean depth	6.275	8.16	5.57	6.215	0.401

Clean reads: the number of high-quality reads; Clean base: the total number of bases of high-quality sequencing data remaining after raw data filtering; Q30(%): the percentage of bases with a quality value greater than or equal to 30 in the clean data set; GC(%): percentage of GC bases among all bases in Clean Data; Present tags: the number of enzyme section segments present in one sample; Present SNPs: identified SNPs of one sample; Mean depth: average depth statistics.

**Figure 2:**
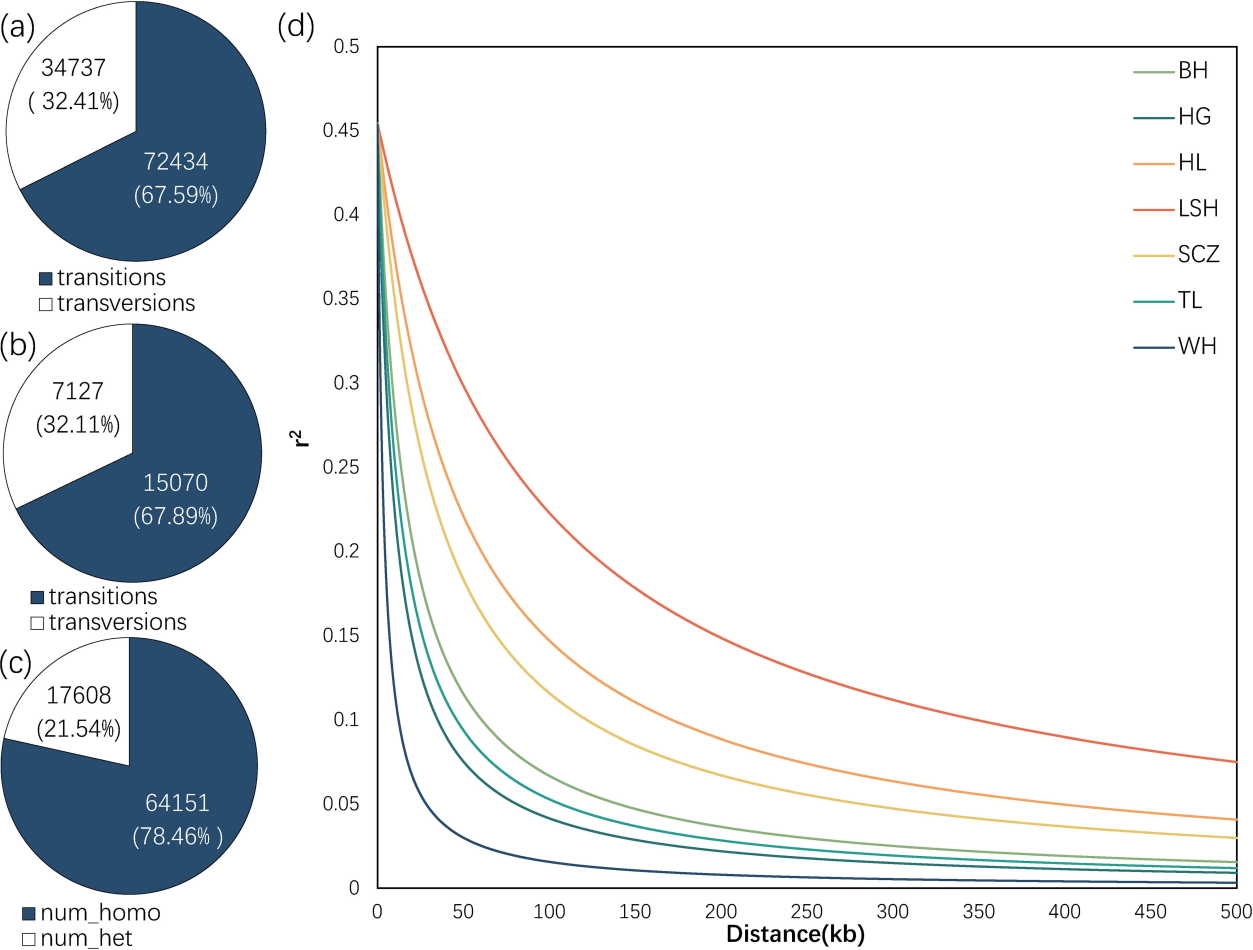
Characterization of filtered variant sites and LD-decay. **(a)** Statistics of the number of variant sites across all samples in the filtered VCF. Transitions: the total number of sites where transitions occur; transversions: the total number of sites where transversions occur. **(b)** Statistics of the mean number of variant sites per sample in the filtered VCF. **(c)** The mean number of genotypes per sample. num_het: the number of heterozygous genotypes; num_homo: the number of homozygous genotypes. **(d)** LD-decay of all populations. Distance: spacing between SNPs. r2: the coefficient of LD. Population abbreviations: BH Bohai, HG Hegang, HL Helong, LSH Lushuihe, SCZ Sanchazi, TL Tieli, WH Weihe.

A total of 32.41% of all the SNPs are transitions, whereas 67.59% are transversions. The mean value for the proportion of transitions among SNPs in each sample is 32.11%, whereas the mean value for transversions is 67.89%. The mean percentages of homozygous genotypes and heterozygous genotypes among the SNPs in each sample were 78.46% and 21.54%, respectively. In the context of LD analysis across different populations, the distance between loci corresponding to half the decay of r² can be employed as a measure of LD-decay. The mean LD-decay among SNP pairs ranged from 3.38 kb (WH) to 96.25 kb (LSH), with an overall mean of 31.40 kb. The observation of broader LD in certain populations may be indicative of a more advanced selection status than in other populations (**Figure 2**).

### 3.2 Genetic diversity and structure

The results of the genetic diversity analysis across populations indicate the following (**Table 3**): BH presented the highest number of unique SNPs, with 5 SNPs. With the exception of LSH (107,162), all other populations presented 107,171 variant sites. The primary reason is that the number of clones in LSH is the smallest, resulting in the absence of a small number of SNPs. WH presented the greatest number of polymorphic sites (103,034) and a polymorphism ratio of 96.104%, whereas LSH presented the lowest number of polymorphic sites (72,026) and a polymorphism ratio of 67.212%. The population with the highest mean number of individuals per site was WH (19), whereas LSH had the lowest (5). This is attributable primarily to the sample size of each population. BH presented the lowest observed heterozygosity (0.205), whereas SCZ presented the highest (0.224). Overall, the observed heterozygosity was comparable to the expected heterozygosity, with the exception of LSH, where the observed heterozygosity was slightly higher than the expected heterozygosity (by only 0.003) and exhibited the lowest inbreeding coefficient (0.058). The remaining populations presented lower observed heterozygosity than expected heterozygosity and higher inbreeding coefficients, suggesting a slight deficiency in heterozygosity. The polymorphism information content (*PIC*) exhibited considerable variation across populations, ranging from 0.176–0.197. The population with the highest Shannon index was WH (0.377), whereas LSH presented the lowest value (0.331). Overall, there were minor differences in the level of genetic diversity among the populations. WH and TL presented greater genetic diversity, whereas LSH presented more scientifically optimized mating combinations.

**Table 3:** Genetic diversity indices of the populations.

Pop ID	Private sites	Total sites	Polymorphic sites	Polymorphic loci/%	Num indv	Ho	He	Fis	PIC	I
BH	5	107171	91063	84.970	11	0.205	0.224	0.107	0.186	0.357
HG	2	107171	97846	91.299	14	0.215	0.231	0.102	0.193	0.370
HL	0	107171	80113	74.753	7	0.213	0.218	0.068	0.179	0.340
LSH	0	107162	72026	67.212	5	0.219	0.216	0.058	0.176	0.331
SCZ	0	107171	87231	81.394	8	0.224	0.230	0.070	0.190	0.362
TL	1	107171	96453	89.999	12	0.223	0.237	0.091	0.197	0.376
WH	1	107171	103034	96.140	19	0.211	0.234	0.126	0.196	0.377

Pop ID: population identification number; Private Sites: number of population-specific sites; Total Sites: number of variant sites; Polymorphic Sites: number of polymorphic sites; Polymorphic Loci/%: percentage of polymorphic sites to total sites; Num Indv: number of individuals in the population containing all sites; Ho: observed heterozygosity; He: expected heterozygosity; Fis: inbreeding coefficient; PIC: polymorphism information content of populations; I: Shannon index of the population.

**Figure 3:**
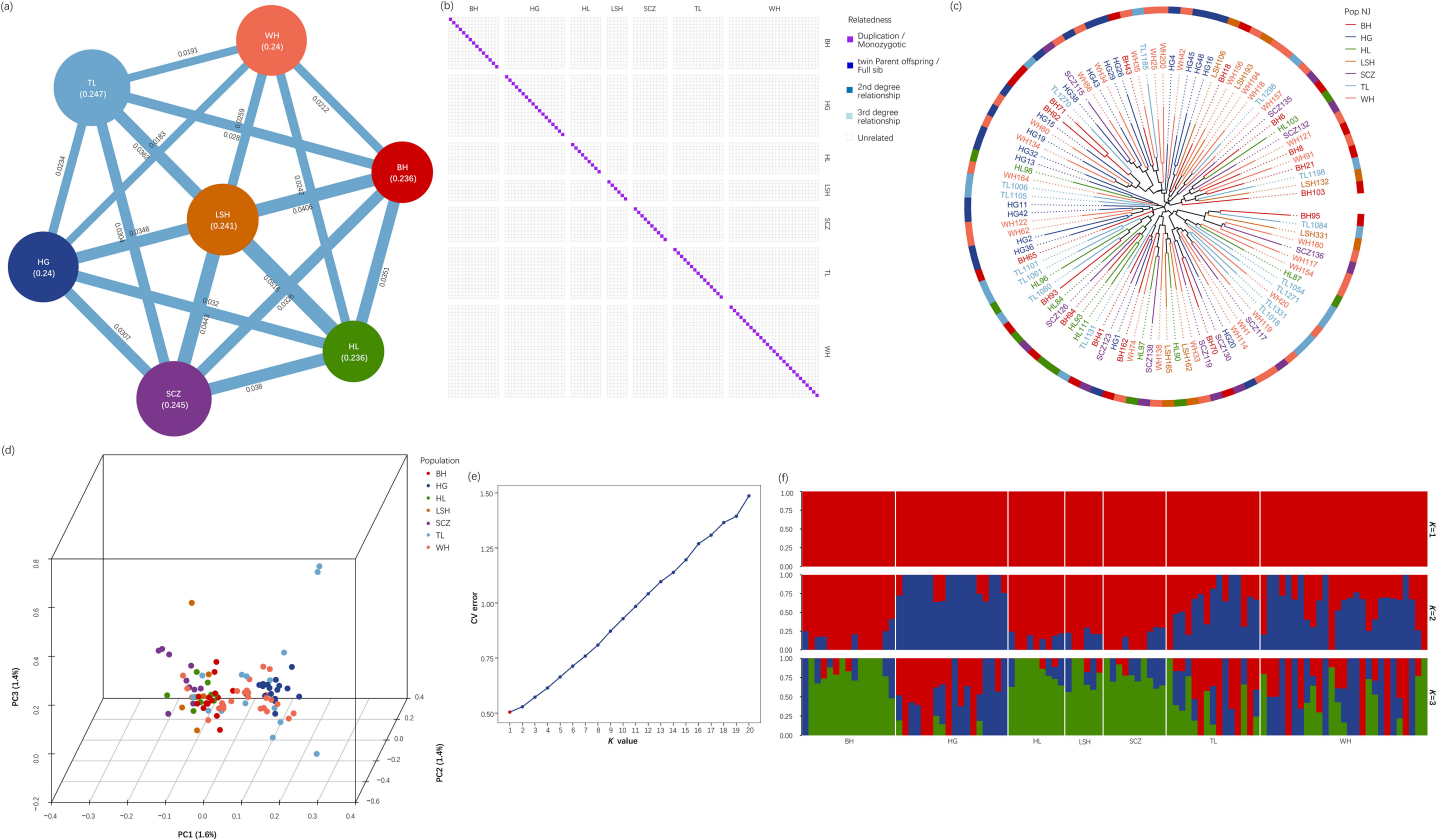
Genetic differentiation and structure analysis. **(a)** Pi among populations and Fst between populations. **(b)** Heatmap of kinship relationships for all individuals. **(c)** Circular evolutionary branch-length plot (NJ). **(d)** PCA based on the first three principal components. **(e)** CV error variation with the K value. **(f)** Genetic components of samples corresponding to the best K value and the nearest K value.

The nucleotide polymorphism (Pi) among populations ranged from 0.236 to 0.247, and the genetic differentiation (Fst) between populations ranged from 0.0183 to 0.0516 (**Figure 3a**), indicating a low level of genetic differentiation among populations and a close genetic relationship among them. This weak inter-population differentiation is consistent with the biological characteristics of Pinus koraiensis: the species features long-distance wind pollination, wind-dispersed pine seeds with moderate migration ability, and continuous large-scale natural forest distribution across Northeast China since the post-glacial period, which promotes frequent gene flow among geographically separated populations and restricts genetic divergence (**Figure 3b**). An evolutionary tree comprising 100 samples was constructed via the NJ method (**Figure 3c**). This revealed no discernible clustering of individuals from different populations on the evolutionary tree. Principal component analysis (PCA) indicated (**Figure 3d**) that all 7 populations presented similar genetic backgrounds, with the first three principal components accounting for 1.6%, 1.4%, and 1.4% of the genetic variance, respectively. The results of the population genetic structure analysis indicated that the most likely number of subpopulations was one. The CV error continuously increased when K > 1, which further verified that all clones belonged to a single ancestral genetic group, so only the top three PCA axes were used to adjust population structure in GWAS rather than ADMIXTURE ancestry components. suggesting the absence of a discernible subpopulation structure (**Figures 3e, f**). This finding was consistent with the results of the preceding analysis.

### 3.3 Phenotypic traits and GWAS

**Figure 4:**
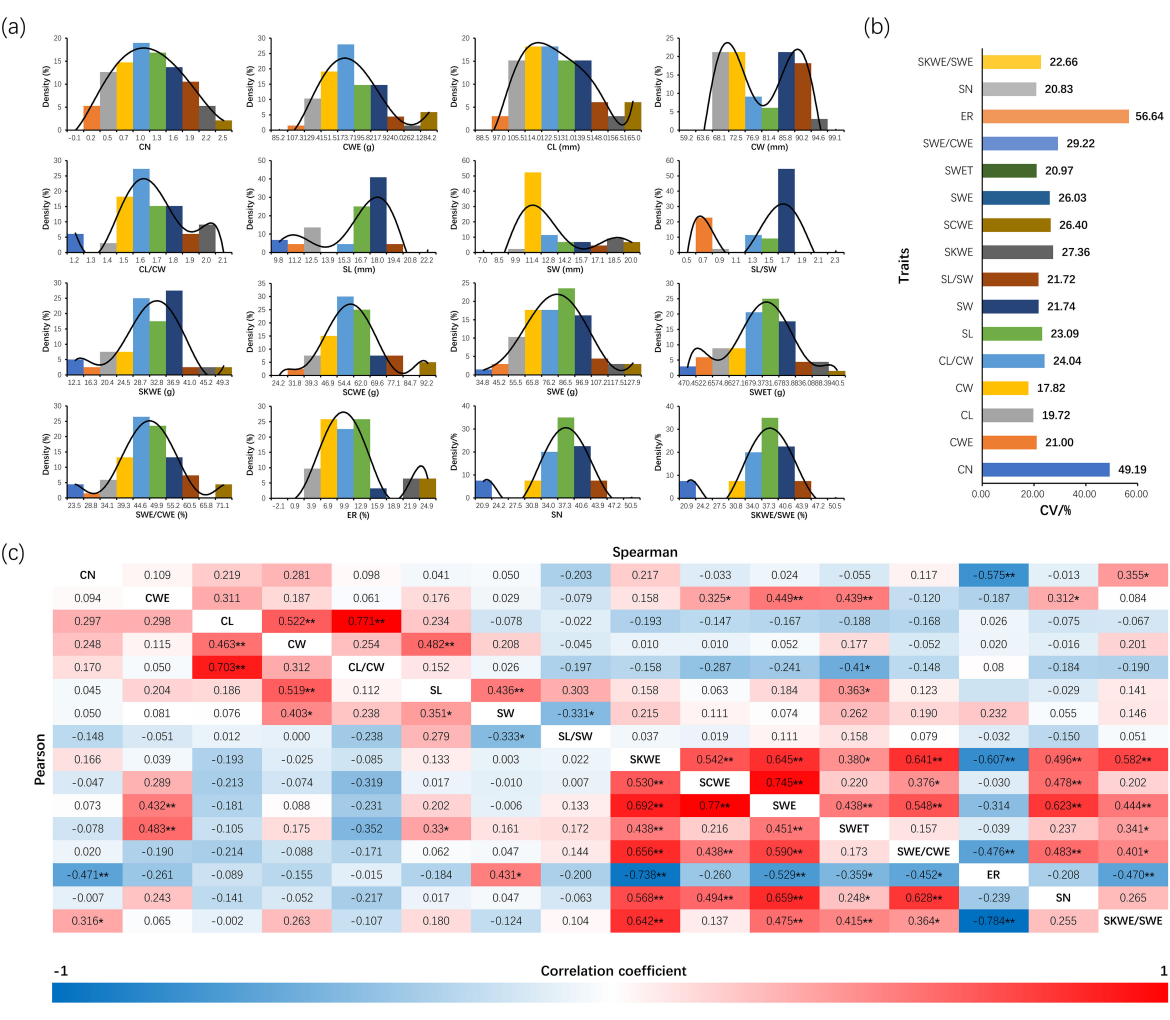
Results of phenotypic trait analysis. **(a)** Distribution test of phenotypic values for each trait; **(b)** analysis of differences and variations among traits; **extremely significant differences among clones (P<0.01). **(c)** correlation analysis of traits. *Significant correlation between traits (P<0.05), **extremely significant correlation between traits (P<0.01).

Initially, distribution tests were conducted on the 16 observed traits, and the results (**Figure 4a**) indicated that all 16 traits exhibited a normal distribution, thereby providing a foundation for subsequent statistical analysis. The variance and variation analyses of each trait among the clones (**Figure 4b**) revealed highly significant differences (P<0.01) in each trait among the clones. The coefficient of variation (CV) for each trait ranged from 17.82% (CW) to 56.64% (ER). The significant variation observed in each trait among the clones provides a robust foundation for subsequent GWASs. Second, the correlations between traits were analysed (**Figure 4c**), and both the Spearman and Pearson methods revealed extremely significant correlations (P<0.01) between 22 and 24 pairs of traits, respectively. This may provide additional sources for GWASs and facilitate the process of assisted breeding on the basis of GWAS results.

**Figure 5:**
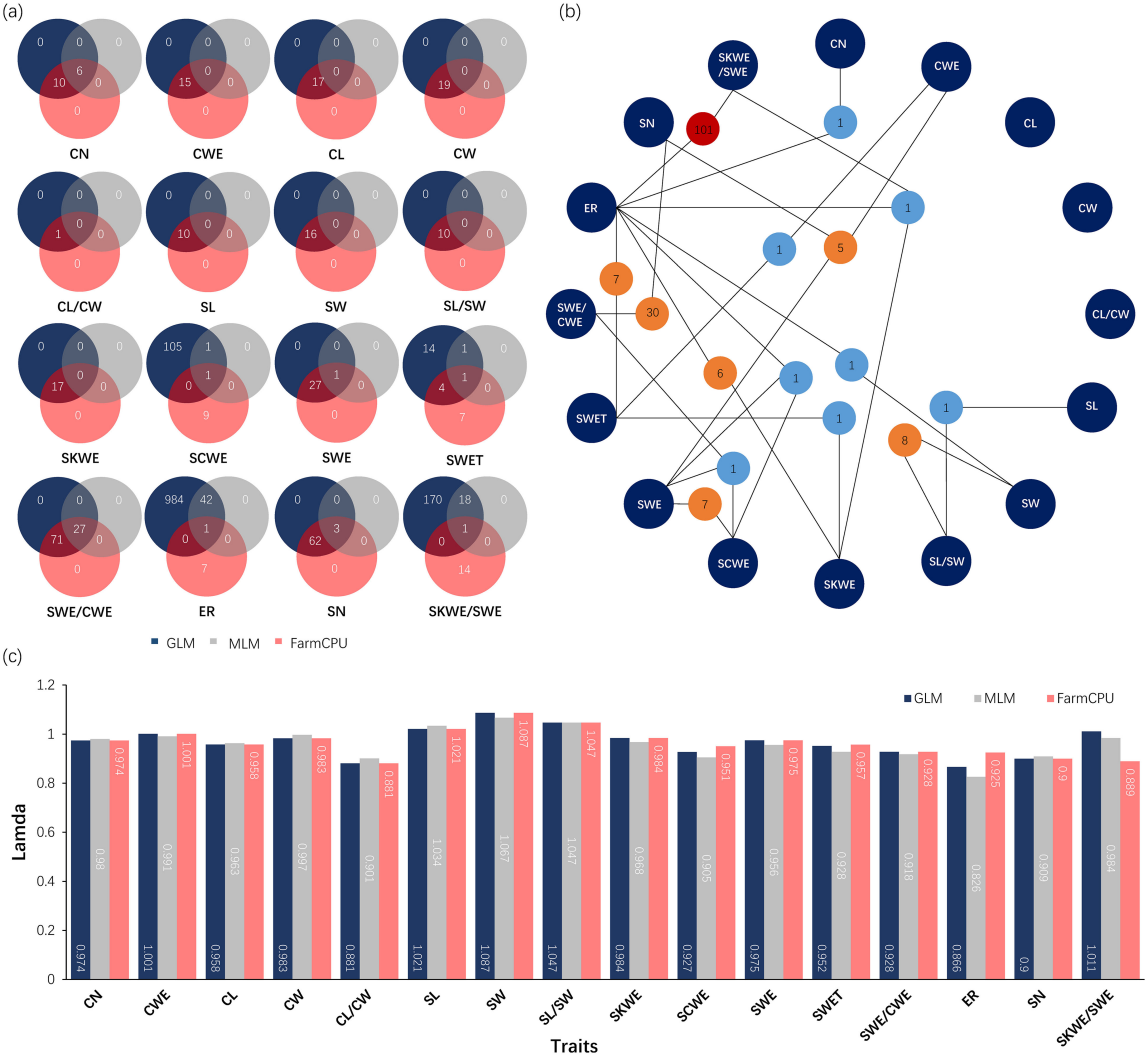
Results of GWAS. **(a)** Venn diagram of the number of SNPs associated with different algorithms for each trait. **(b)** Network diagram of the shared SNPs associated with each trait. **(c)** Lambda values corresponding to the various algorithms for each trait.

**Figure 5a** shows the number of SNPs associated with different traits according to various algorithms. The ER is associated with the largest number of SNPs; however, only one SNP is commonly associated across the three algorithms. Conversely, CL/CW is associated with the fewest SNPs, with only one SNP being associated. A total of 214 pleiotropic SNPs were identified, which simultaneously associated with two or more economic traits. The pleiotropic loci shared by ER and SKWE/SWE reached the maximum number (101), representing core genetic regions regulating seed quality traits. These multi-trait associated SNPs are ideal molecular targets for synchronous improvement of multiple yield traits in Korean pine breeding programs (**Figure 5b**). **Figure 5c** shows the inflation factor lambda for each trait under the three algorithms. The majority of the trait lambda values deviate from 1 by less than 0.1. The lambda deviation is more pronounced for ER, which is associated with the greatest number of SNPs, and CL/CW, which is associated with the lowest number of SNPs. This suggests the possibility of false-positive results due to population stratification in these two traits.

### 3.4 Development of KASP

KASP primers were designed for the SNPs associated with two traits, CN and CWE. KASP marker primers, Pk1780727, Pk4881799, Pk494448, and Pk3270935, were successfully designed for each trait. The primer information is presented in **Table 4**.

**Table 4:** Information on the KASP primers.

Trait	SNP	Primer	Sequence (5’–3’)
CN	Pk1780727	F1	GAAGGTGACCAAGTTCATGCTTGAAAACTTGCAATGTTGTAACTGC
		F2	GAAGGTCGGAGTCAACGGATTCTTGAAAACTTGCAATGTTGTAACTGT
		R	TTTTGGCAATGCGATGAGGTC
	Pk4881799	F1	GAAGGTGACCAAGTTCATGCTTCAGTTTTACCCCCCGCCGATC
		F2	GAAGGTCGGAGTCAACGGATTTCAGTTTTACCCCCCGCCGATT
		R	GGACACGTGGACGGCCCAAATG
CWE	Pk494448	F1	GAAGGTGACCAAGTTCATGCTATCTCTTACAGTCCCAGTTATGAAATTA
		F2	GAAGGTCGGAGTCAACGGATTATCTCTTACAGTCCCAGTTATGAAATTG
		R	TGCTGAAGGTGGATCATTTGGTT
	Pk3270935	F1	GAAGGTGACCAAGTTCATGCTGAAAGTACAAAGGTTGAAGTGTTTCAG
		F2	GAAGGTCGGAGTCAACGGATTGAAAGTACAAAGGTTGAAGTGTTTCAT
		R	TGAAGGACATATGCACCTCAGAAA

**Figure 6:**
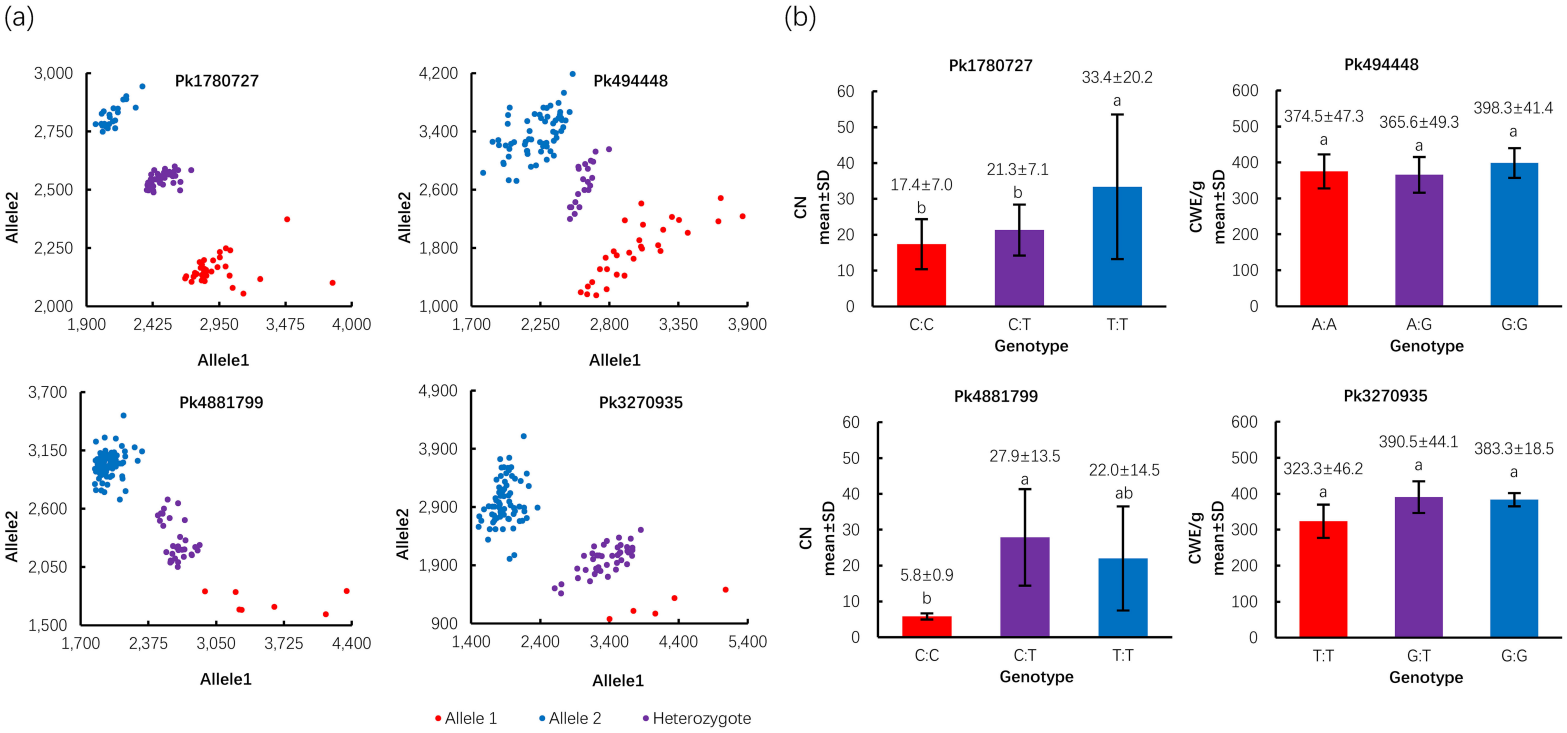
Information on relevant SNPs and KASP validation results. **(a)** Genotyping maps of natural Korean pine plus trees from Xiaobeihu. **(b)** Multiple comparisons (Duncan) of traits associated with genotype groups for each KASP marker.

The genotyping results of natural Korean pine plus trees from Xiaobeihu, obtained via synthesis KASP primers, are presented in **Figure 6a**. A statistical analysis was conducted on the observed data from consecutive years for the related traits on the basis of the genotyping results of the aforementioned trees (**Figure 6b**). The results demonstrated that the mean CN of the homozygous mutant (T:T) at Pk1780727 was 33.4, which was notably greater than that of the heterozygous mutant (C:T) (21.3) and the wild type (C:C) (17.4). For Pk4881799, the mean CN of the homozygous mutant (T:T) and heterozygous mutant (C:T) strains were 22 and 27.9, respectively, which were markedly greater than that of the wild-type (C:C) strain (5.8). Furthermore, there was a notable disparity between the heterozygous mutant (C:T) and the wild type (C:C). The mean CWE of the homozygous mutant (G:G) at Pk494448 was 398.3 g, which was greater than that of the wild type (A:A) (374.5 g) and the heterozygous mutant (A:G) (365.6 g). With respect to Pk3270935, the mean CWE of the heterozygous mutant (G:T) and homozygous mutant (G:G) strains are 390.5 g and 383.3 g, respectively, which are markedly greater than that of the wild type (T:T) strain (323.3 g). After eliminating environmental heterogeneity via linear mixed model correction, mutant genotypes of Pk494448 and Pk3270935 still exhibited numerically higher adjusted CWE values than wild-type genotypes without significant statistical differences (P>0.05).

## 4 Discussion

This study conducted genetic analysis within clone populations of Korean pine via RAD-Seq technology, successfully implementing a GWAS and developing KASP markers. This effort not only deepened our understanding of the genetic diversity within Korean pine, but also provided powerful tools for subsequent genetic breeding programs and forest management practices. First, RAD-Seq technology has demonstrated its ability to capture high-density SNPs efficiently across the genome. Sequencing the Korean pine clones generated rich genetic variation data that comprehensively covered the entire genome, providing a solid foundation for subsequent genetic analyses and GWASs. The density and uniform distribution of SNPs ensured the accuracy and reliability of the genetic analyses. The genetic analysis revealed that the different populations had comparable levels of genetic diversity, shared similar genetic backgrounds with minimal genetic differentiation and no discernible substructure, which is consistent with our previous findings from studies of natural populations (Yan et al., 2024a). In contrast to previous low-density SSR-based diversity analysis (Yan et al., 2023) which only captured dozens of polymorphic loci, this RAD-Seq study generated over 14 million genome-wide SNP markers, providing saturated genome variation information. High-density RAD-SNPs exhibited stronger power to dissect subtle population stratification and capture minor-effect variants associated with yield economic traits, whereas SSR markers were limited to neutral genetic diversity evaluation and failed to mine trait-causal loci. The high-throughput nature of this technology allows a wider range of genetic information to be captured, improving the precision and depth of genetic analysis. The high-density SNPs obtained in this study have greater explanatory power, elucidating the genetic basis of the Korean pine germplasm.

Our team previously established a Korean pine core collection containing 114 clones via SSR markers and conifer traits (Yan et al., 2024b). However, the primary objective of core germplasm collections is to conserve genetic diversity, which relies mainly on pedigree analysis, genetic markers and quality traits (Charmet et al., 1993; Gepts, 2006). As Korean pine is an important edible nut species (Du et al., 2017), yield-related traits are often of paramount importance in selection. Therefore, this study used GWAS to uncover several SNPs significantly associated with important economic traits of Korean pine, including CN and CWE. Among these, CN, our primary focus, was associated with 6 relevant SNPs consistently identified by three algorithms. This finding offers the potential to improve the breeding efficiency of Korean pine through MAS in the future, and provides important clues for subsequent functional gene research and breeding practices.

KASP, known for its high specificity, sensitivity and low cost, has been widely used in plant genetics and breeding, including in crops such as rice (*Oryza sativa*), soybean (*Phaseolus vulgaris* L.), Chinese cabbage (*Brassica rapa* L. ssp. *pekinensis*) and wheat (*Triticum aestivum* L.) (Singh et al., 2019; Yang et al., 2020; Zaleski-Cox et al., 2023; Sandhu et al., 2024). However, the development of KASP markers for Korean pine faces challenges due to the lack of a reference genome and the presence of multiple SNPs within a single read. Despite these obstacles, 4 KASP markers were successfully developed on the basis of GWAS results for CN and CWE, allowing rapid, accurate and efficient genotyping of the associated SNPs. When these markers were applied to genotype-based groupings of natural Korean pine plus trees, significant differences in associated traits between groups were observed for CN-associated SNPs. For SNPs associated with CWE, homozygous or heterozygous mutations tended to be more common than in the wild type, although this difference was not statistically significant. This may be because CWE is a quantitative trait under complex genetic control and is susceptible to environmental factors. Natural populations lack strict environmental control and there is considerable variation in tree age, nutritional status and site conditions, all of which contribute to this phenomenon. Compared with traditional molecular markers, KASP markers offer advantages such as simplicity, low cost and reliability, making them more suitable for large-scale genetic breeding practices. As a result, they provide an effective means for the rapid identification and evaluation of Korean pine germplasm resources. To further verify the universality of CWE-related KASP markers in breeding practice, multi-site validation experiments will be carried out in three independent seed orchards (Bohai, Tieli and Hegang) in follow-up research, using grafted clones with consistent age and cultivation management to avoid environmental interference and confirm marker stability.

Despite significant advances in the genetic analysis of Korean pine, GWAS and the development of KASP markers in this study, several limitations remain. First, the sample size of the present study was limited to 100 clones derived from only six seed orchards in Heilongjiang and Jilin provinces, lacking germplasm resources from Liaoning provenances and marginal natural populations, which cannot fully represent the whole genetic variation range of natural Korean pine populations across Northeast China. For follow-up research, more provenance clones covering all main distribution areas of Korean pine should be collected to expand the germplasm panel and improve the universality of population genomics and GWAS results. Second, the study focused mainly on phenotypic traits, with insufficient correlation analysis for other important traits. In addition, the natural superior tree population used to validate the KASP markers lacked experimental treatments and strict environmental control. On the basis of the results and limitations of this study, we propose the following recommendations for future research: (1) Increasing the number of Korean pine provenances can provide a more comprehensive view of genetic diversity within the population, thereby improving the accuracy and reliability of genetic analyses. (2) GWASs on Korean pine seed components such as oil, protein and amino acids can be performed. (3) KASP markers can be validated through extensive field trials and data analysis to further confirm their stability and reliability, and evaluate their practical application in Korean pine breeding. (4) RAD-Seq technology can be integrated with multiomics approaches such as transcriptomics and proteomics to elucidate the mechanisms of genetic and phenotypic variation in Korean pine from multiple perspectives and provide more comprehensive theoretical guidance for breeding practices. (5) On the basis of the GWAS results and KASP markers, functional gene research can be conducted to reveal the functions and regulatory mechanisms of genes associated with critical traits in Korean pine, thus providing precise molecular targets for breeding practices. (6) Due to the absence of high-quality *Pinus koraiensis* reference genome, direct functional annotation of GWAS-associated SNPs cannot be realized in the present study. As a feasible follow-up strategy, all RAD tags carrying significant CN/CWE-related SNPs will be mapped to published homologous conifer genomes via sequence blast. Conserved orthologous genes within mapped syntenic blocks will be screened as candidate genes controlling cone yield traits, providing fundamental sequence resources for subsequent gene function verification.

The genetic diversity of Korean pine populations is similar, and there is little differentiation between populations. Several SNPs related to economic traits, such as CN, have been obtained through GWAS. The developed KASP markers promote MAS in breeding programs, improving the efficiency and accuracy of genetic improvement in Korean pine. The research results provide a new technique for understanding the genetic structure of Korean pine and offer a powerful tool for targeted breeding and genetic improvement.

## Data Availability

The datasets generated and/or analysed during the current study are available in the Genome Se-quence Archive (Chen et al., 2021a) in National Genomics Data Center (Xue et al., 2022), China Na-tional Center for Bioinformation/Beijing Institute of Genomics, Chinese Academy of Sciences repository, CRA021625.
